# Digital violence against women and children: the state of the literature

**DOI:** 10.3389/fsoc.2026.1977287

**Published:** 2026-10-21

**Authors:** Thendo Phophi Mbambala, Mampilo Phahlane

**Affiliations:** Department of Information Systems, CSET School of Computing, University of South Africa (UNISA), Pretoria, South Africa

**Keywords:** children, digital violence, justice gap, online harms, platform governance, thematic synthesis, women

## Abstract

**Purpose:**

This state-of-the-literature review synthesizes interdisciplinary scholarship on digital violence affecting women and children, with the aim of clarifying dominant harm typologies, socio-technical drivers, evidence practices, and governance responses. The review responds to increasing global concern regarding technology-facilitated violence and persistent justice gaps in prevention, reporting, and redress.

**Design/methodology/approach:**

The review adopts a state-of-the-literature design combining transparent literature identification and screening with reflexive thematic synthesis. Search was conducted across Scopus, Web of Science Core Collection, EBSCOHost, ProQuest, Google Scholar, and selected policy sources, covering publications from January 2000 to March 2026. A PRISMA-style flow diagram is used to ensure transparency. The analysis does not claim protocol-driven systematic review status.

**Findings:**

The literature conceptualizes digital violence as persistent, boundary-eroding, and structurally enabled through platform affordances, algorithmic amplification, and data-driven political economies. Evidence is constrained by fragmented definitions, inconsistent measurement practices, and geographic concentration in the Global North. These limitations contribute to recurring justice gaps across legal systems, platform governance, and support mechanisms.

**Originality/value:**

The article consolidates a fragmented body of scholarship into an integrated analytical framework encompassing domain, structural, epistemological, and socio-political dimensions. It identifies literature-grounded priorities for regulation, platform accountability, prevention, survivor-centered support, and future research.

## 1 Introduction

Digital technologies increasingly mediate education, work, social interaction, and civic participation. Alongside these benefits, however, a growing body of research documents how digital environments enable and intensify harms that disproportionately affect women and children. International policy bodies now recognize technology-facilitated violence as a significant and escalating gendered and intergenerational risk (Antoninis et al., 2023; UN Women, 2025).

A defining feature of digital violence is its persistence and capacity for boundary erosion. Harms can be reproduced, amplified, and re-circulated across time and platforms, often extending into offline consequences that reshape victims’ participation, safety practices, and access to opportunities (Henry and Powell, 2018; Harris and Vitis, 2020). Platform architectures characterized by scalability, anonymity, and virality further complicate containment and accountability (Gillespie, 2018; Zuboff, 2024).

Despite extensive scholarly attention, the evidence base remains fragmented across disciplines, methods, and regions. Definitions vary widely, measurement practices are inconsistent, and Global South experiences are under-represented. These limitations constrain coherent policy responses and contribute to ongoing justice gaps across legal systems and platform governance arrangements (Czerniewicz et al., 2020; Antoninis et al., 2023; Polyzoidou, 2024).

### 1.1 Objectives and research questions

The objective is to consolidate and interpret current scholarship to clarify what is known, how it is known, and what remains unresolved. The review addresses RQ1–RQ4 covering domain, structural, epistemological and socio-political dimensions (Polyzoidou, 2024; Díaz-Pérez et al., 2026; Coombs, 2021).

1. RQ1 (domain): how are digital harms against women and children defined and classified across disciplines?
2. RQ2 (structural): what socio-technical drivers (e.g., platform affordances, algorithms, incentives) enable or amplify these harms?
3. RQ3 (epistemological): how are harms evidenced and measured, and what limits and biases shape the available evidence?
4. RQ4 (socio-political): how do legal, policy, and platform governance responses operate, and where do justice gaps persist?

## 2 Rationale and contribution

This review responds to conceptual fragmentation, methodological inconsistency, and geographic imbalance in the literature on digital violence. While individual studies offer valuable insights, the absence of integrative synthesis limits cumulative knowledge and policy relevance (Jewkes and Dartnall, 2019). By consolidating scholarship across criminology, gender studies, education, governance, and platform studies, the review contributes a structured thematic framework, and a consolidated map of evidence gaps to support future research and policy development (Henry and Powell, 2018; Antoninis et al., 2023).

The urgency of this synthesis is underscored by three factors. First, the frequency and complexity of digital violence are escalating, with recent global surveys indicating that 73% of women have experienced some form of online violence (UN Women, 2025). Second, legal and policy responses consistently lag technological change, creating a “justice gap” that leaves survivors without adequate recourse (Polyzoidou, 2024; Coombs, 2021). Third, the interdisciplinary nature of the field means that insights remain siloed, limiting the development of comprehensive, evidence-informed interventions (Bailey et al., 2021; Powell and Flynn, 2023).

## 3 Methodology

This study employed a state-of-the-literature review design to map and critically interpret scholarship on digital violence against women and children. PRISMA 2020 (Page et al., 2021) guided the transparent reporting of identification, screening, eligibility, and inclusion, while selected SPAR-4-SLR (Paul et al., 2021) structured the organization and documentation of the review process. Reflexive thematic synthesis (Thomas and Harden, 2008; Braun and Clarke, 2006) informed the interpretive development of descriptive and analytical themes. The review was not registered as a systematic-review protocol and does not claim the exhaustive coverage or effect-estimation objectives associated with a meta-analysis.

### 3.1 Literature identification

Searches were conducted in Scopus, Web of Science Core Collection, EBSCOHost, and ProQuest, supplemented by Google Scholar and targeted policy sources from the United Nations, World Health Organization, and UN Women. The search covered publications from January 2000 to March 2026, reflecting the period during which digital violence emerged as a distinct field of scholarly inquiry.

Search strings combined keywords relating to digital violence, including “technology-facilitated violence,” “online abuse,” “cyberbullying,” “digital harassment,” “image-based abuse,” “women,” and “children.” Boolean operators were used to refine results across databases.

### 3.2 Screening and corpus

The screening and study-selection process was documented using the PRISMA 2020 reporting structure (Page et al., 2021) and comprised five broad phases: identification, deduplication, title-and-abstract screening, full-text eligibility assessment, and final inclusion (see Table 1).

**Table 1:** Screening process.

Stage	Description	Count
Identification	Records identified through database searching	512
Deduplication	Records after duplicates removed	428
Screening	Records screened by title and abstract	428
Title/Abstract exclusion	Records excluded at title/abstract stage	293
Eligibility	Full-text articles assessed for eligibility	135
Full-text exclusion	Records excluded at full-text stage	77
Included	Sources included in final corpus	58

Exclusion reasons at full-text stage (see Table 2):

**Table 2:** Reasons for full-text exclusion.

Reason	Count
Insufficient focus on women and/or children	18
Primarily technical focus without social/policy analysis	15
Duplicate publication of the same data	12
Offline violence without digital dimension	11
Inadequate methodological rigor	9
Not available in English	7
Opinion piece lacking scholarly grounding	5
Total excluded	77

The screening process yielded: identified (*n* = 512), after duplicates removed (*n* = 428), screened (*n* = 428), excluded at title/abstract (*n* = 293), full texts assessed (*n* = 135), excluded at full text (*n* = 77), included in final corpus (*n* = 58) (see Figure 1).

**Figure 1:**
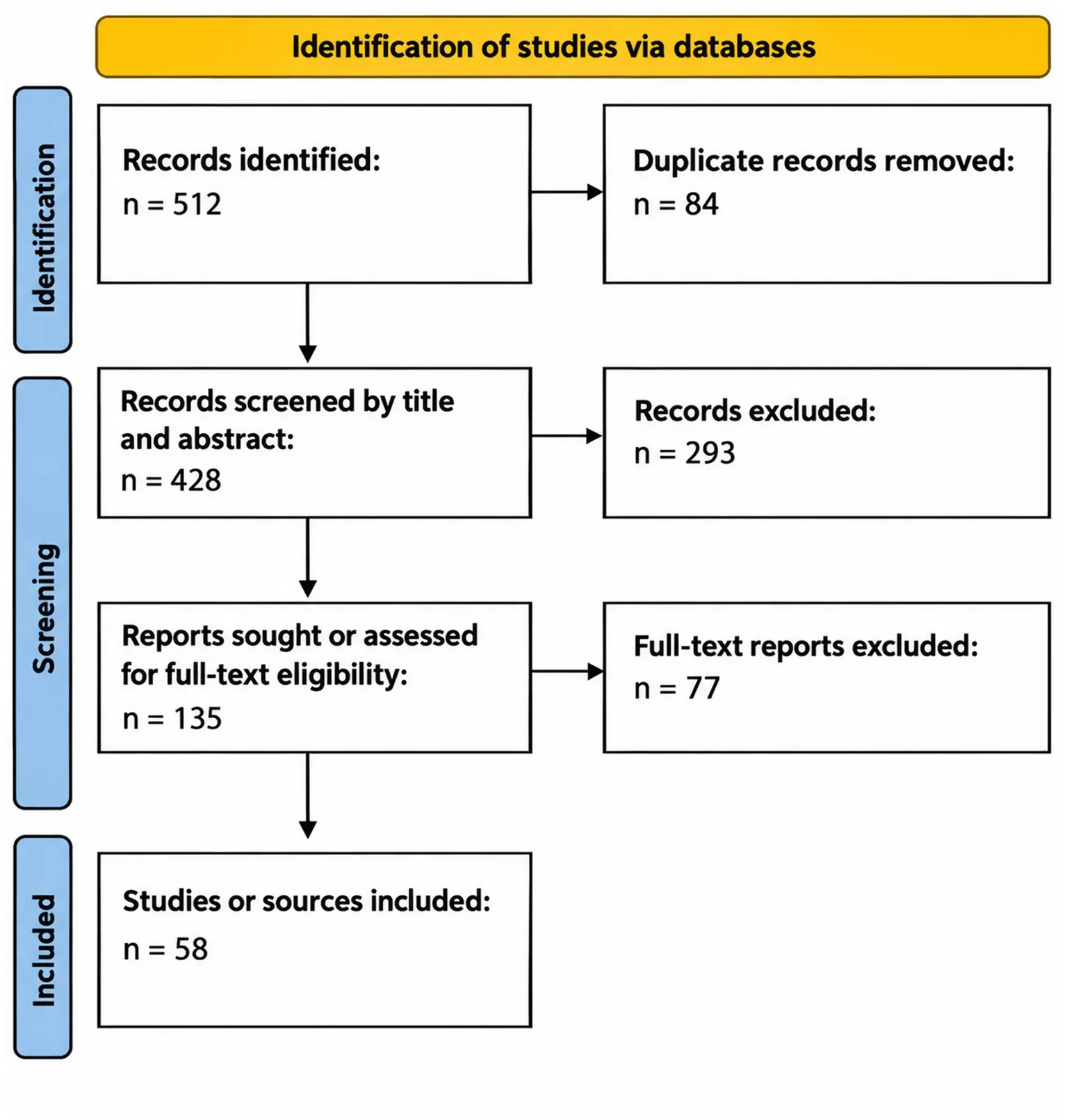
PRISMA flow diagram of study selection (*n* = 58).

### 3.3 Quality appraisal

Because the corpus included heterogeneous empirical, conceptual, review, and policy sources, a single formal risk-of-bias instrument was not applied across all source types. Sources were critically considered in relation to their relevance to the research questions, transparency of purpose and method, coherence between evidence and conclusions, theoretical contribution, and value to the synthesis. These considerations informed interpretation rather than serving as a citation-based ranking or exclusion mechanism.

### 3.4 Reflexive thematic synthesis

The analysis employed thematic synthesis (Thomas and Harden, 2008), a method specifically designed for synthesizing qualitative research and conceptual literature in systematic reviews. The approach involves three stages: (1) line-by-line coding of source material, (2) organization of initial codes into descriptive themes, and (3) development of analytical themes that go beyond the original studies.

#### 3.4.1 Coding process

Two authors independently coded a subset of sources; disagreements were resolved through discussion. Formal inter-coder agreement statistics were not calculated because the analysis was interpretive and iterative (see Table 3).

**Table 3:** Coding process.

Stage	Output
Familiarization	Reading notes and preliminary observations
Initial coding	1,247 initial codes
Code consolidation	247 consolidated codes or categories
Descriptive-theme development	27 descriptive themes
Analytical theme development	4 analytical themes aligned with RQ1–RQ4

#### 3.4.2 Theme development

Themes were developed iteratively through four rounds of review and refinement:

1. Round 1: Initial coding and descriptive theme generation.
2. Round 2: Two authors reviewed the descriptive themes and resolved differences through discussion.
3. Round 3: Mapping descriptive themes to research questions.
4. Round 4: Refining analytical themes and checking against the full dataset.

### 3.5 Corpus of included sources

The following table presents the 58 sources included in the final corpus, organized by author and year, region, discipline, method, focus, and key contribution.

The Corpus reflects the primary regional focus of each source. Sources with multi-country, conceptual or global scope are classified as cross-national/global. Methodological references (e.g., Braun and Clarke, 2006; Page et al., 2021; Paul et al., 2021; Thomas and Harden, 2008) are not included in this corpus table (see Table 4).

**Table 4:** Characteristics of included sources.

Author (year)	Region/Context	Discipline/Lens	Method/Type	Focus	Key contribution
Antoninis et al. (2023)	Global	Education/Policy	Report	Technology in education	Flags gendered online risk
Baekgaard (2024)	Global	Policy/Gender	Report	TFGBV	Evidence stocktake
Bailey et al. (2021)	Global	Interdisciplinary	Edited volume	TFVA	Comprehensive international handbook
Bates (2017)	United Kingdom	Criminology	Qualitative	Image abuse	Victim impact
Bucher (2018)	Global	Media studies	Conceptual	Algorithms	Analyses algorithmic influence
Castaño-Pulgarín et al. (2021)	Global	Criminology	Systematic review	Online hate	Synthesizes cyberhate
Citron (2014)	United States	Law	Legal analysis	Cyber harassment	Cyber civil rights model
Coombs (2021)	United States	Socio-legal	Journal article	Digital justice	Introduces justice gap concept
Davidson and Gottschalk (2011)	Global	Criminology	Book	Cybercrime	Examines exploitation risks
Díaz-Pérez et al. (2026)	Latin America	Law/Regulation	Journal article	Cross-border enforcement	Highlights jurisdictional fragmentation
Douglas et al. (2019)	Australia	Criminology	Qualitative interviews	IPV digital abuse	Survivor experiences
Dragiewicz et al. (2018)	Global	Feminist media	Qualitative	Coercive control	Defines TF coercive control
Enock et al. (2024)	Global	Computational social science	Journal article	Evidence practices	Highlights methodological constraints
Flynn and Henry (2021)	Australia	Criminology	Journal article	Image-based abuse	Australian reflection
Freed et al. (2017)	United States	Security studies	Qualitative	Surveillance abuse	Spyware use in IPV
Gill (2007)	Global	Media studies	Conceptual	Gender media	Explains cultural inequality
Gillespie (2018)	Global	Media studies	Scholarly monograph	Content moderation	Frames moderation as governance shaping visibility of harm
Ging and Siapera (2018)	Europe	Media studies	Edited volume	Online misogyny	Cultural dynamics
Grossman et al. (2024)	Global	Policy	Report	Child safety ecosystem	Strengths and weaknesses
Harris and Vitis (2020)	Global	Gender-based violence; criminology	Conceptual article	Digital intrusions, spatiality	Explains persistence and boundary erosion of harm
Henry and Powell (2015)	Australia	Criminology	Qualitative	Digital abuse	Early evidence
Henry and Powell (2018)	Australia/United Kingdom	Criminology; feminist law	Systematic review	TFSV	Foundational synthesis of prevalence and impacts
Henry et al. (2020)	Global	Gender studies	Review	Domestic digital violence	Expands TFSV framework
Holt and Bossler (2014)	United States	Criminology	Quantitative	Cybercrime	Provides victimization data
Hrick (2021)	Global	Policy	Book chapter	Survivor-centered responses	Centralized approaches
Im et al. (2022)	United States	HCI/Platform studies	Conference paper	Reporting behaviors	Shows how reporting shapes justice outcomes
Jane (2014)	Australia	Media studies	Qualitative	Online misogyny	Defines “e-bile”
Jewkes and Dartnall (2019)	Global	Public health	Commentary	Research gaps	Calls for further research
Kelly (2013)	United Kingdom	Feminist theory	Conceptual	GBV continuum	Foundational theory
Kowalski et al. (2014)	USA	Psychology	Review	Cyberbullying	Behavioral impacts
Livingstone and Stoilova (2021)	Global	Childhood studies	Conceptual	Online risk to children	4C’s classification
Mantilla (2013)	United States	Gender studies	Conceptual	Gender harassment	Defines cyber misogyny
Marganski and Melander (2021)	Global	Criminology	Book chapter	TFVA justice	Moving from enemy to ally
Marwick and Caplan (2018)	United States	Media studies	Qualitative	Networked harassment	Coordinated abuse
Masenya (2023a)	Africa	Feminist/Intersectionality	Journal article	Inequality	Centers intersectional vulnerability
Masenya (2023b)	South Africa	Gender studies	Journal article	Gender inequality	Theoretical discriminatory perspective
McGlynn et al. (2017)	United Kingdom	Law	Conceptual	Image-based abuse	Continuum theory
Mohanty (2003)	Global South	Feminist theory	Conceptual	Postcolonial feminism	Challenges western bias
Noble (2018)	United States	Critical theory	Book	Algorithms of oppression	Highlights algorithmic bias
Olaitan (2024)	South Africa	Development studies	Journal article	GBV and technology	Adds global south perspective
Patchin and Hinduja (2015)	United States	Criminology	Quantitative	Cyberbullying	Identifies risk factors
Polyzoidou (2024)	Global	Socio-legal	Journal article	Criminalization	Explains persistence of justice gaps
Powell et al. (2020)	Australia	Criminology	Survey	Victimization	Empirical prevalence
Powell (2023)	Global	Criminology	Book chapter	Technology-facilitated abuse	IPV in digital society
Powell and Flynn (2023)	Global	Feminist criminology	Journal article	TFVA victimization	Gendered analysis
Roberts (2019)	Global	Media studies	Ethnographic	Moderation	Reveals hidden labor
Salter and Blodgett (2017)	Global	Criminology	Conceptual	Digital crime	Links cyber abuse systems
Strathern and Pfeffer (2022)	Global	Computational	Preprint	Online misogyny	Identifying layers of misogyny
Stevens et al. (2024)	Global	Social indicators	Journal article	Measuring harms	Identifies definitional and measurement limits
Suzor (2019)	Global	Law	Legal analysis	Platform governance	Identifies regulatory gaps
Toro-Alvarez (2024)	Global	Education	Journal article	Cyberbullying	Unified theory of digital violence
(UN Women and World Health Organization, 2023)	Global	Policy	Report	Governance responses	Frames institutional response gaps
UN Women (2025)	Global	Gender policy	Report	Digital GBV	Emphasizes scale and policy urgency
Wajcman (2010)	Global	Sociology	Conceptual	Technology and gender	Feminist tech critique
Woodlock (2017)	Australia	Social work	Qualitative	IPV tech abuse	Abuse escalation
Walby (2013)	Global	Sociology	Conceptual	Violence measurement	Provides comparison framework
Whittle et al. (2013)	United Kingdom	Criminology	Qualitative	Grooming	Identifies offender strategies
World Health Organization (2022a,b)	Global	Public health	WHO report	Prevention	Positions digital violence as public health issue
Zuboff (2024)	Global	Political economy	Scholarly monograph	Datafication, surveillance	Links platform incentives to harm amplification

#### 3.5.1 Geographic distribution of the corpus

Of the 58 sources included in the final corpus, 22 (37.9%) focused primarily on Global North contexts, while four (6.9%) focused principally on Global South contexts. The remaining 32 sources (55.2%) were classified as cross-national, global, conceptual, theoretical, review, or policy-oriented works without a single identifiable empirical setting. Among region-specific sources, Global North contexts were substantially more represented than Global South contexts (see Table 5).

**Table 5:** Geographic distribution of included sources.

Region/Context	Number of sources	Percentages
Global North (United States, United Kingdom, Europe, Australia/NZ)	22	37.9%
Global South (Africa, Latin America, Asia, Middle East)	4	6.9%
Cross-national/Global	32	55.2%
Total	58	100%

### 3.6 Methodological limitations

The methodology has several limitations that affect the generalizability of findings:

*Language restriction*: Inclusion of only English-language publications introduces linguistic bias and may exclude important contributions from non-English speaking contexts.

*Geographic imbalance*: The search strategy and database coverage favor Global North publications, limiting representation of Global South perspectives despite efforts to include them.

*Publication bias*: The emphasis on peer-reviewed sources may exclude important grey literature, practitioner reports, and community-generated knowledge.

*Temporal constraints*: The 2000–March 2026 timeframe may exclude foundational works and may not capture the most recent developments given the rapidly evolving nature of the field.

*Interpretive nature*: Thematic synthesis involves researcher interpretation, which, while reflexive, cannot claim objectivity.

## 4 Findings

### 4.1 Domain: definitions and typologies (RQ1)

Across disciplines, digital violence is conceptualized as a continuum of harms encompassing harassment, intimidation, image-based abuse, surveillance, and reputational damage (Henry and Powell, 2018; Harris and Vitis, 2020). However, there is limited conceptual alignment regarding the boundaries and classification of these harms. Legal and criminological scholarship tends to categorize digital violence through discrete offense-based frameworks, such as cyberstalking or image-based abuse (Henry and Powell, 2018). In contrast, feminist and socio-cultural scholarship emphasizes the fluidity, persistence, and interconnected nature of harms across online and offline contexts (Harris and Vitis, 2020; McGlynn et al., 2017).

#### 4.1.1 Typologies of digital violence

This divergence produces significant analytical and policy challenges. While offense-based approaches support legal clarity, they often fail to capture the cumulative and escalating nature of harm. Conversely, continuum-based approaches better reflect lived experiences but complicate measurement and legal categorization (Polyzoidou, 2024) (see Table 6).

**Table 6:** Typologies of digital violence.

Category	Description	Examples	Key sources
Technology-facilitated sexual violence (TFSV)	Non-consensual sexual acts or threats enabled by technology	NCII, sextortion, cyber-flashing, sexual extortion	Henry and Powell (2018); McGlynn et al. (2017)
Digital coercive control	Use of technology to surveil, isolate, and control intimate partners	Spyware, password control, location tracking, excessive monitoring	Dragiewicz et al. (2018); Woodlock (2017)
Gendered online harassment	Gender-based abuse targeting women and girls	Cyberstalking, doxing, trolling, misogynistic abuse	Jane (2014); Mantilla (2013); Marwick and Caplan (2018)
Child-specific exploitation	Abuse uniquely targeting children	Grooming, CSAM production/distribution, peer-based harassment	Whittle et al. (2013); Livingstone and Stoilova (2021)
Image-based abuse	Non-consensual creation, sharing, or threat of sharing intimate images	Revenge porn, deepfakes, sextortion	McGlynn et al. (2017), Bates (2017), Flynn and Henry (2021)

A recurring concept across the literature is boundary erosion, whereby harms are not confined to a single platform or moment but persist, migrate, and intensify across temporal and spatial contexts (Harris and Vitis, 2020). This erosion manifests in three ways:

1. Spatial: Harms cross from online to offline, entering victims’ homes and workplaces.
2. Temporal: Abusive content persists indefinitely, creating ongoing vulnerability.
3. Jurisdictional: Harms cross national boundaries, complicating legal response.

Despite broad agreement that digital violence is multidimensional, definitional fragmentation continues to limit comparative research, obscure prevalence estimates, and weaken policy coherence. This lack of conceptual clarity remains a central constraint in advancing both research standardization and effective intervention.

#### 4.1.2 Tensions in the literature

The review identified three key tensions in how digital violence is defined and typologized:

##### 4.1.2.1 Tension 1: offense-based vs. continuum frameworks

This divergence produces significant analytical and policy challenges. While offense-based approaches support legal clarity, they often fail to capture the cumulative and escalating nature of harm. Conversely, continuum-based approaches better reflect lived experiences but complicate measurement and legal categorization (Polyzoidou, 2024). The literature reveals an unresolved debate about whether digital violence requires special criminalization (creating new offenses specifically for online harms) or adaptation of existing laws (extending current legal frameworks to cover digital contexts) (Polyzoidou, 2024; Citron, 2014).

##### 4.1.2.2 Tension 2: technology-specific vs. context-specific approaches

Some scholars focus on the unique features of digital environments (anonymity, scalability, persistence), while others emphasize that digital violence is fundamentally an extension of offline violence. The former risks technological determinism; the latter risks obscuring how technology transforms the nature and experience of violence. Harris and Vitis (2020) navigate this tension through the concept of boundary erosion, showing how digital harms are both continuous with offline violence and qualitatively transformed by technological affordances.

##### 4.1.2.3 Tension 3: universal vs. culturally specific definitions

Global North definitions of digital violence may not align with how harms are experienced and understood in other cultural contexts. Mohanty (2003) and Olaitan (2024) highlight how universal frameworks risk imposing Western assumptions about gender, violence, and technology on contexts where power relations and cultural norms differ significantly. Masenya (2023a) argues for intersectional approaches that center the experiences of women in the Global South, challenging the dominance of Western feminist frameworks.

### 4.2 Structural: socio-technical drivers (RQ2)

The literature consistently demonstrates that digital violence is not incidental but structurally enabled by platform architectures and political-economic incentives. Platform affordances such as anonymity, scalability, and virality facilitate both the reach and persistence of abusive content (Gillespie, 2018). At the same time, algorithmic amplification and engagement-driven business models prioritize visibility and interaction, often at the expense of user safety (Zuboff, 2024; Noble, 2018).

#### 4.2.1 Critical debates on structure and agency

##### 4.2.1.1 Debate 1: unintended consequences vs. designed outcomes

While some scholarship frames platform dynamics as unintended consequences of technological innovation (Gillespie, 2018), an emerging body of work argues that platform design actively reproduces and intensifies pre-existing inequalities, particularly gendered and generational power imbalances. From this perspective, harms are not simply user-generated but are co-produced through technological systems that reward attention, controversy, and emotional engagement (Zuboff, 2024; Noble, 2018). The evidence increasingly supports the latter view, with multiple studies showing how engagement metrics systematically disadvantage marginalized users (Gillespie, 2018; Roberts, 2019; Bucher, 2018).

##### 4.2.1.2 Debate 2: platform accountability

The literature reveals significant disagreement about the extent to which platforms can or should be held accountable for harms. Some scholars argue for strong regulatory frameworks that mandate safety-by-design and impose liability for platform-facilitated harms (Suzor, 2019; Citron, 2014). Others warn against over-regulation that may stifle innovation or enable state surveillance (Zuboff, 2024). This debate reflects deeper tensions between free expression, user protection, and the political economy of digital platforms.

#### 4.2.2 Three interconnected structural dynamics

##### 4.2.2.1 Digitized reproduction of patriarchal power

Digital violence is not a new phenomenon but the digital resurfacing of pre-existing hierarchies of power. Patriarchal domination of women’s bodies and sexuality is mirrored and amplified in digital spaces, with perpetrators employing technological affordances to exercise gendered norms and punish violations of those norms (Citron, 2014; Masenya, 2023a,b). This insight is critical: digital violence is not a separate phenomenon but an extension of structural inequality.

##### 4.2.2.2 Platform capitalism and harm amplification

The basic structure and business models of digital platforms enable and amplify harm through:

*Surveillance capitalism*: The data extractive economy provides materials (location data, personal information) that can be weaponized for stalking and harassment (Zuboff, 2024).

*Algorithmic amplification*: Social media algorithms prioritize engagement, allowing abusive content to be amplified quickly and allowing hate groups to connect (Gillespie, 2018; Noble, 2018).

Anonymity and pseudonymity: While valued by some users, these features allow perpetrators to more easily engage in abuse and place severe barriers on accountability (Marwick and Caplan, 2018).

##### 4.2.2.3 Generational and adultism dynamics

Children’s vulnerability to digital violence is compounded by adultist assumptions that minimize their experiences and limit their agency. Platforms designed for adult users often fail to provide adequate protections for younger users, while legal frameworks may not recognize the severity of peer-to-peer harms (Livingstone and Stoilova, 2021; Grossman et al., 2024). This creates a particularly acute vulnerability for children and adolescents.

##### 4.2.2.4 Implications

The evidence strongly supports the view that harm is structurally enabled rather than merely incidental. Responsibility extends beyond individual perpetrators to platform design, governance logics, and political-economic incentives. This has significant implications for intervention: effective responses must address structural conditions rather than focusing solely on individual behavior or post-hoc moderation.

### 4.3 Epistemological: evidence and measurement (RQ3)

The production of evidence on digital violence is characterized by significant methodological and epistemological challenges. Across literature, inconsistent definitions and indicators limit comparability between studies and hinder the development of cumulative knowledge (Stevens et al., 2024; Enock et al., 2024). Quantitative research often struggles to capture context-specific experiences of harm, while qualitative studies provide depth but are limited in generalizability (Henry and Powell, 2018).

#### 4.3.1 Critical analysis of evidence production

##### 4.3.1.1 The problem of under-reporting

A consistent finding is the prevalence of underreporting, which is frequently interpreted as a rational response to low trust in institutional and platform-based reporting mechanisms. Survivor narratives indicate that reporting can be time-consuming, emotionally burdensome, and unlikely to result in meaningful outcomes (Im et al., 2022). As a result, official statistics often underestimate the scale and severity of digital violence.

##### 4.3.1.2 Epistemic injustice

The literature also highlights forms of epistemic injustice, whereby survivor knowledge is marginalized or devalued within formal systems of evidence production. This is particularly evident in contexts where experiential accounts are dismissed as subjective or insufficiently verifiable (Masenya, 2023a; Polyzoidou, 2024). For example, non-consensual image sharing is often legally interpreted as a privacy violation rather than a form of sexual violence, diminishing the nature of that violence and making it difficult to prosecute (de Araújo et al., 2022; McGlynn et al., 2017).

##### 4.3.1.3 Geographic knowledge imbalance

“Among sources with a clearly identifiable regional focus, Global North contexts were substantially more represented than Global South contexts.” This imbalance creates interventions that may not be meaningful to regional contexts, given different legal systems, resource limitations, and cultural conditions (Olaitan, 2024; Masenya, 2023a). The literature from the Global South that does exist highlights:

#### 4.3.2 Key epistemological constraints

Different cultural and social dynamics affecting how violence is experienced and reported (Olaitan, 2024) (see Table 7).

**Table 7:** Epistemological constraints.

Constraint	Description	Evidence	Key sources
Inconsistent definitions	No agreed-upon definitions across studies	Different concepts operationalized; incomparable findings	Stevens et al. (2024); Enock et al. (2024)
Under-reporting	Low trust in institutions; fear of victim-blaming	Official statistics underestimate prevalence	Im et al. (2022); Henry and Powell (2018)
Epistemic injustice	Survivor knowledge marginalized or devalued	Experiential accounts dismissed as subjective	Masenya (2023a); Polyzoidou (2024)
Geographic bias	Over-representation of global north	Findings may not apply to other contexts	Olaitan (2024); Czerniewicz et al. (2020)
Methodological fragmentation	Disciplinary silos	Limited cross-disciplinary integration	Powell and Flynn (2023); Bailey et al. (2021)

Resource constraints affecting both research capacity and intervention (Masenya, 2023b).

Colonial legacies affecting knowledge production and policy development (Mohanty, 2003).

Taken together, these constraints produce a fragmented and uneven evidence base that weakens accountability, limits policy effectiveness, and obscures the lived realities of those most affected.

### 4.4 Socio-political: governance and justice gaps (RQ4)

Governance responses to digital violence remain fragmented across legal systems, platform regulation, and policy frameworks. While there is growing recognition of technology-facilitated violence as a policy issue, implementation remains inconsistent and often reactive. Legal frameworks frequently struggle to keep pace with technological change, resulting in gaps in protection, enforcement, and redress (Polyzoidou, 2024; UN Women and World Health Organization (2023)).

#### 4.4.1 Key justice gaps identified

A central theme across the literature is the persistence of justice gaps, defined as the disconnect between harm experienced and remedy available (Coombs, 2021). These gaps are shaped by multiple factors (see Table 8):

**Table 8:** Justice gaps.

Gap	Description	Mechanism	Key sources
Jurisdictional complexity	Cross-border nature of internet	Difficulty determining applicable law and enforcement	Polyzoidou (2024); Díaz-Pérez et al. (2026)
Platform self-regulation limits	Inconsistent content moderation	Uneven enforcement; burden on users	Gillespie (2018); Roberts (2019)
Evidentiary challenges	Difficulty collecting and preserving evidence	Prosecution barriers; victim-blaming	Henry and Powell (2018); UN Women and World Health Organization (2023)
Institutional cultures	Victim-blaming; lack of training	Under-reporting; secondary victimization	Coombs (2021); Grossman et al. (2024)
Gendered assumptions	Legal frameworks shaped by male experience	Harms experienced by women not recognized	Citron (2014); Wajcman (2010)

##### 4.4.1.1 Jurisdictional complexity

The cross-border reach of the internet renders difficult the decisions on which laws are applicable and which enforcement agency is responsible. This complexity creates “safe havens” for perpetrators who operate across jurisdictions (Polyzoidou, 2024; Díaz-Pérez et al., 2026).

##### 4.4.1.2 Ineffective platform governance

Although platform governance mechanisms such as reporting systems and content moderation policies have expanded, they are often criticized for lack of transparency, inconsistency in enforcement, and limited responsiveness to user needs (Gillespie, 2018; Roberts, 2019). Survivor experiences suggest that existing mechanisms frequently shift the burden of safety onto individuals rather than addressing structural drivers of harm (Im et al., 2022).

##### 4.4.1.3 Institutional and systemic barriers

*Victim-blaming cultures*: Fear of being disbelieved or blamed, combined with a lack of trained law enforcement, leads to substantial under-reporting (Coombs, 2021).

*Inconsistent enforcement*: Even when laws exist, enforcement is uneven, particularly for low-level harms (Polyzoidou, 2024).

*Barriers to reporting*: Lack of clear, accessible reporting mechanisms; fear of reprisal; lack of trust in authorities (Im et al., 2022)

##### 4.4.1.4 Institutional and systemic barriers

At the same time, there is evidence of emerging alternative approaches:

*Survivor-led advocacy*: Survivor-led organizations and hashtag activism have shifted public perception, exerted influence on systems, and built solidarity and community (Bailey et al., 2021).

*Community-based interventions*: Grassroots efforts to create alternative support networks and digital self-defense resources (Olaitan, 2024).

*Policy innovation*: National and international efforts to push for effective legislative reform, including the EU Digital Services Act (UN Women and World Health Organization, 2023).

*Technological resistance*: Development of secure communication apps and alternative platforms (Olaitan, 2024).

However, these initiatives often operate without sufficient institutional support or scalability, limiting their broader impact (Grossman et al., 2024; Hrick, 2021).

Governance responses to digital violence remain fragmented across legal systems, platform regulation, and policy frameworks. While there is growing recognition of technology-facilitated violence as a policy issue, implementation remains inconsistent and often reactive. Legal frameworks frequently struggle to keep pace with technological change, resulting in gaps in protection, enforcement, and redress (Polyzoidou, 2024).

A central theme across the literature is the persistence of justice gaps, defined as the disconnect between harm experienced and remedy available. These gaps are shaped by multiple factors, including jurisdictional fragmentation, limitations of platform self-regulation, evidentiary challenges, and institutional cultures that may reinforce victim-blaming attitudes.

Although platform governance mechanisms such as reporting systems and content moderation policies have expanded, they are often criticized for lack of transparency, inconsistency in enforcement, and limited responsiveness to user needs. Survivor experiences suggest that existing mechanisms frequently shift the burden of safety onto individuals rather than addressing structural drivers of harm.

At the same time, there is evidence of emerging alternative approaches, including survivor-led advocacy, community-based interventions, and policy innovation. However, these initiatives often operate without sufficient institutional support or scalability, limiting their broader impact.

### 4.5 Cross-cutting synthesis: structural harm and evidence inequality

Across all themes, the literature converges on the understanding that digital violence against women and children is structurally embedded within socio-technical systems rather than isolated or incidental. Platform design, governance limitations, and fragmented evidence practices interact to sustain conditions that enable harm.

At the same time, there is a persistent lack of integration across conceptual, empirical, and policy-oriented research. This fragmentation contributes directly to ongoing justice gaps and weakens the coherence of responses across sectors. The overrepresentation of Global North contexts further exacerbates these issues, as policy frameworks and interventions are often developed without sufficient consideration of context-specific constraints in underrepresented regions (see Table 9).

**Table 9:** Thematic synthesis.

Research question	Core theme	Sub-themes	Representative sources	Implications
RQ1: domain—definitions and typologies	Digital violence as a continuum of harms	• Harassment and intimidation • Image-based abuse • Digitally enabled surveillance • Boundary erosion between online/offline harm	Harris and Vitis (2020); Im et al. (2022); Citron (2014); McGlynn et al. (2017)	Fragmented definitions hinder measurement, cross-study comparison, and legal categorization; policy and research require conceptually coherent frameworks that recognize persistence and harm escalation across platforms
RQ2: structural—socio-technical drivers	Digitized reproduction of structural inequalities	• Platform affordances (anonymity, scalability, virality) • Algorithmic amplification • Engagement-driven business models • Patriarchal and adultist power relations	Gillespie (2018); Zuboff (2024); Masenya (2023a,b); Powell and Flynn (2023); Strathern and Pfeffer (2022)	Harm is not accidental but structurally enabled; responsibility extends beyond individual perpetrators to platform design, governance logics, and political-economic incentives.
RQ3: epistemological—evidence and measurement	Epistemic injustice and evidence fragility	• Inconsistent definitions and indicators • Under-reporting and trust deficits • Marginalization of survivor knowledge • Geographic and disciplinary bias	Enock et al. (2024); Stevens et al. (2024); Henry and Powell (2018); UN Women and World Health Organization, 2023)	Weak and uneven evidence constrains effective intervention; future research must integrate survivor-centered, context-sensitive, and methodologically plural approaches.
RQ4: socio-political—governance and justice gaps	Fragmented governance and justice deficits	• Jurisdictional fragmentation • Platform self-regulation limits • Inconsistent enforcement • Barriers to reporting and redress	Polyzoidou (2024); Díaz-Pérez et al. (2026); Coombs (2021); Grossman et al. (2024)	Justice gaps persist due to misalignment between law, policy, and platform regulation; coordinated, cross-sector governance and trauma-informed procedures are needed.
Cross-cutting theme (all RQs)	Evidence inequity and Global South marginalization	• Over-representation of Global North research • Limited contextualization in low-resource settings • Policy transfers without local fit	Olaitan (2024); Masenya (2023a); UN Women (2025))	Research and policy agendas must address geographic imbalance to prevent the reproduction of digital injustice through one-size-fits-all interventions.

Addressing digital violence therefore requires not only improved regulation and institutional responses but also greater alignment between research, policy, and practice. Without such integration, interventions risk reproducing the very inequalities they seek to address.

## 5 Discussion

Synthesizing across the four research questions demonstrates that digital violence against women and children is not incidental but structurally embedded within socio-technical systems. Fragmented definitions, fragile evidence practices, and uneven governance capacity interact to sustain justice gaps. Effective responses therefore require coordinated interventions spanning legal reform, platform accountability, prevention, and survivor-centered support.

### 5.1 The interplay between domain, structural, epistemological, and socio-political dimensions

The four dimensions are analytically interconnected rather than independent. Definitional fragmentation under RQ1 produces inconsistent indicators and weak comparability under RQ3. These evidentiary weaknesses make it difficult for legal and institutional systems to recognize cumulative or cross-platform harms, thereby reinforcing the justice gaps identified under RQ4. At the same time, the platform architectures and political-economic incentives examined under RQ2 shape both the production of harm and the evidence available to document it. The result is a reinforcing cycle in which structurally enabled harms are inconsistently defined, incompletely measured, and inadequately governed.

#### 5.1.1 Key insights from synthesis

##### 5.1.1.1 Digital violence as structural, not incidental

The review demonstrates that digital violence is not a series of isolated incidents or the result of individual bad actors. Rather, it is systematically enabled by platform architectures, business models, and governance failures. This challenges approaches that focus solely on individual perpetrators and calls for systemic responses (Zuboff, 2024; Gillespie, 2018).

##### 5.1.1.2 The persistence of justice gaps

Despite growing awareness of the problem, justice gaps persist across jurisdictions. These gaps are not merely technical or legal but reflect deeper institutional and structural failures, including (see Table 10):

**Table 10:** Justice gaps.

Failure type	Manifestation	Key sources
Legal	Frameworks designed for offline violence applied inappropriately	Polyzoidou (2024); Citron (2014)
Governance	Platform governance prioritizes profitability over safety	Gillespie (2018); Zuboff (2024)
Institutional	Cultures reinforce victim-blaming	Coombs (2021); Im et al. (2022)
Resource	Lack of resources for investigation and prosecution	Grossman et al. (2024); (UN Women and World Health Organization, 2023)

##### 5.1.1.3 The centrality of evidence equity

The review highlights the importance of addressing geographic and epistemological biases in research. Without evidence that reflects the experiences of diverse populations, interventions risk reproducing the inequalities they seek to address (Olaitan, 2024; Mohanty, 2003; Czerniewicz et al., 2020).

##### 5.1.1.4 The need for cross-sector coordination

Effective responses require coordination across legal systems, platform governance, education, and support services. No single sector can address the problem alone. This coordination must be informed by survivor-centered, trauma-informed approaches (World Health Organization, 2022a,b; UN Women and World Health Organization, 2023).

##### 5.1.1.5 The potential of emergent responses

The review also identifies promising developments, including survivor-led advocacy, policy innovation, and community-based interventions. However, these initiatives require institutional support and scaling to achieve broader impact.

### 5.2 Contradictions and debates in literature

The review identified several unresolved debates. First, whether digital violence requires special criminalization or adaptation of existing laws. Second, how far platforms can or should be held accountable for harms. Third, whether responses should be given to upstream prevention or downstream response. These debates reflect broader tensions between legal clarity and lived experience, innovation and regulation, universalism and contextualism, and prevention versus redress. Resolving them requires comparative, context-sensitive evidence (see Table 11).

**Table 11:** Contradictions and debates in literature.

Debate	Positions	Evidence	Implications
Special criminalization vs. adaptation	Some argue for new laws specifically addressing digital violence; others advocate adapting existing laws to digital contexts.	Mixed evidence; depends on legal culture	Affects legislative strategy and implementation
Platform accountability	Varying views on regulatory mechanisms and enforcement	Limited evidence on effectiveness of different approaches	Affects governance approaches
Global vs. local responses	Tension between universal frameworks and culturally specific approaches	Strong evidence for contextual variation	Affects policy transfer and implementation
Prevention vs. response	Different emphasis on upstream prevention vs. downstream support	Limited comparative evidence	Affects resource allocation

### 5.3 Research implications

The synthesis suggests several directions for future research:

#### 5.3.1 Conceptual integration

Need for integrated frameworks that bridge offense-based and continuum-based approaches, while remaining attentive to cultural and contextual variations. This requires interdisciplinary dialog and collaborative research.

#### 5.3.2 Methodological innovation

Need for pluralistic, mixed method approaches that combine quantitative prevalence studies with qualitative understanding of lived experience. Need for standardized measurement tools that are culturally validated across contexts.

#### 5.3.3 Geographic diversification

Urgent need for research in the Global South that is context-sensitive, participatory, and attentive to local cultural, legal, and resource conditions. Need for research capacity building in underrepresented regions.

#### 5.3.4 Governance research

Need for evidence on what governance mechanisms work, in what contexts, and for whom. Need for comparative research across jurisdictions and regulatory approaches. Need for research on implementation and enforcement, not just policy design.

#### 5.3.5 Survivor-centered research

Need for participatory methodologies that center survivor knowledge and agency. Need for longitudinal research on impacts and recovery. Need for research on the effectiveness of support services and interventions.

### 5.4 Policy and practice implications

The findings suggest that effective responses to digital violence require interventions that extend beyond individual behavior and address the structural conditions that enable harm. Across the literature, legal reform, platform accountability, prevention strategies, survivor-centered support systems, and evidence-informed policymaking emerge as mutually reinforcing priorities. However, the effectiveness of any single intervention is likely to remain limited unless coordinated across governance, technological, educational, and community contexts. The recommendations outlined in Section 8 translate these broader implications into specific actions for policymakers, platform providers, practitioners, and researchers (Gillespie, 2018; Zuboff, 2024; Bailey et al., 2021; World Health Organization, 2022a,b; UN Women and World Health Organization, 2023).

## 6 Limitations

As a state-of-the-literature review, findings are shaped by the scope and accessibility of published sources. Several limitations warrant acknowledgment:

### 6.1 Language and geographic limitations

The review includes only English-language publications, potentially excluding important scholarship from non-English speaking contexts. Despite efforts to include Global South perspectives, among sources with a clearly identifiable regional focus, Global North contexts were substantially more represented than Global South contexts, which may limit the applicability of findings to underrepresented regions. This limitation is both methodological and epistemic: it reflects and reinforces the geographic imbalance identified in literature.

### 6.2 Corpus limitations

While the review draws on 58 sources, this corpus reflects the scope and accessibility of published literature rather than the full universe of relevant research. Grey literature, practitioner reports, and community-generated knowledge may be underrepresented. The corpus also reflects the search strategy’s focus on academic databases, which may not capture all relevant policy and practitioner contributions.

### 6.3 Temporal limitations

The search period (2000–March 2026) may exclude foundational works that established key concepts in the field. It may also fail to capture the most recent developments given the rapidly evolving nature of digital technologies and harm. The field’s dynamism means that findings require ongoing updating.

### 6.4 Interpretive limitations

Thematic synthesis is interpretive, and the analytical themes reflect the authors’ theoretically informed engagement with the corpus. Collaborative review and discussion strengthened the transparency and coherence of theme development but did not eliminate researcher subjectivity. Other researchers may interpret or organize the same literature differently.

### 6.5 Publication bias

The emphasis on peer-reviewed sources may exclude important grey literature, practitioner reports, and community-generated knowledge. This may bias findings toward academic perspectives and away from practice-based knowledge. The inclusion of policy reports partially addresses this limitation.

## 7 Conclusion

Digital violence affecting women and children is persistent, boundary-eroding, and structurally enabled by contemporary platform ecosystems. Addressing these harms requires coordinated, evidence-informed governance that aligns legal frameworks, platform design, prevention strategies, and resourced support systems (World Health Organization, 2022a,b; Antoninis et al., 2023).

The review demonstrates that digital violence is not incidental or isolated but embedded within socio-technical systems that systematically enable harm. Fragmented definitions, fragile evidence practices, and uneven governance capacity interact to sustain justice gaps. Effective responses require coordinated interventions spanning legal reform, platform accountability, prevention, and survivor-centered support.

The persistence of justice gaps across jurisdictions indicates that current responses are inadequate. Legal frameworks lag technological change, platform governance is inconsistent and opaque, and support services are under-resourced. Survivor knowledge is often marginalized, and Global South perspectives are underrepresented in the evidence base.

However, the literature also identifies promising developments: survivor-led advocacy, policy innovation, and community-based interventions offer models for more effective responses. The challenge is to move from isolated innovation to systemic transformation, scaling what works while addressing structural inequalities.

The contribution of this review lies in synthesizing a fragmented field into an integrated analytical framework. By consolidating scholarship across disciplines, regions, and methods, the review provides a comprehensive map of current knowledge and identifies priority areas for future research and intervention. The framework encompasses domain, structural, epistemological, and socio-political dimensions, offering a holistic understanding of the problem and its solutions.

## 8 Recommendations

Drawing on the synthesis of evidence, five priorities emerge for addressing digital violence against women and children. First, legal and policy frameworks should provide clearer definitions, stronger cross-border cooperation, and trauma-informed procedures that improve accountability and access to remedies. Second, platform governance should prioritize safety-by-design, transparency, and age-appropriate protections, particularly for children and other vulnerable users. Third, prevention strategies should combine digital literacy, bystander engagement, and institutional responsibility across educational, community, and digital environments. Fourth, support pathways require greater resourcing and coordination to improve reporting processes, survivor support, and access to redress. Finally, future research should address evidence inequities through culturally sensitive, survivor-centered, and geographically diverse approaches that strengthen the quality and applicability of knowledge (Polyzoidou, 2024; Gillespie, 2018; Zuboff, 2024; Livingstone and Stoilova, 2021; Bailey et al., 2021; Olaitan, 2024; UN Women and World Health Organization, 2023).

## Data Availability

The original contributions presented in the study are included in the article/supplementary material, further inquiries can be directed to the corresponding author.
